# Associations of Prenatal Exposure to Phthalates with Measures of Cognition in 4.5-Month-Old Infants

**DOI:** 10.3390/ijerph18041838

**Published:** 2021-02-13

**Authors:** Francheska M. Merced-Nieves, Kelsey L. C. Dzwilewski, Andrea Aguiar, Salma Musaad, Susan A. Korrick, Susan L. Schantz

**Affiliations:** 1 Neuroscience Program, University of Illinois at Urbana-Champaign, Urbana, IL 61801, USA; kelsey.dzw@gmail.com (K.L.C.D.); schantz@illinois.edu (S.L.S.); 2 Beckman Institute for Advanced Science and Technology, University of Illinois at Urbana-Champaign, Urbana, IL 61801, USA; aaguiar@illinois.edu; 3 Department of Environmental Medicine and Public Health, Icahn School of Medicine at Mount Sinai, New York, NY 10029, USA; 4 Department of Comparative Biosciences, University of Illinois at Urbana-Champaign, Urbana, IL 61802, USA; 5 USDA/ARS Children’s Nutrition Research Center, Baylor College of Medicine, Houston, TX 77030, USA; salma.musaad@bcm.edu; 6 Channing Division of Network Medicine, Department of Medicine, Brigham and Women’s Hospital and Harvard Medical School, Boston, MA 02115, USA; susan.korrick@channing.harvard.edu; 7 Department of Environmental Health, Harvard T.H. Chan School of Public Health, Boston, MA 02115, USA

**Keywords:** neurodevelopment, phthalates, sexually dimorphic

## Abstract

The association of prenatal phthalate exposure with physical reasoning was assessed in 159 (78 female; 81 male) 4.5-month-old infants from a prospective cohort. Phthalate metabolites were quantified in urine from 16–18 gestational weeks and a pool of five urines from across pregnancy. Infants’ looking times to physically impossible and possible events were recorded via infrared eye-tracking. Infants that recognize that one of the events is impossible will look at that event longer. Associations of phthalate biomarkers with looking time differences (impossible–possible) were adjusted for maternal age, infant sex, and order of event presentation, and effect modification by infant sex was assessed. Each interquartile range (IQR) increase of monoethyl phthalate in the pooled sample was associated with females’ increased looking time (β = 1.0; 95%CI = 0.3, 1.7 s) to the impossible event. However, for males, an IQR increase in monoethyl phthalate at 16–18 weeks (β = −2.5; 95%CI = −4.4,−0.6 s), the sum of di(isononyl) phthalate metabolites in the pooled sample (β = −1.0; 95%CI = −1.8, −0.1 s), and the sum of all phthalate metabolites in both samples (β = −2.3; 95%CI = −4.4, −0.2 s) were associated with increased looking to the possible event, suggesting that higher prenatal phthalate exposure is associated with poorer physical reasoning in male infants.

## 1. Introduction

Phthalates are endocrine-disrupting chemicals found in a wide range of consumer products from food packaging to personal care products [1]. Phthalates have very short half-lives and exposure varies greatly across time within individuals [1]. However, due to their ubiquitous nature, daily exposure occurs via multiple routes including ingestion, dermal contact, and inhalation [2,3]. Previous studies have found associations between prenatal phthalate exposure and adverse neurodevelopment in multiple domains including cognition, behavior, and motor development [3,4,5,6,7,8].

Furthermore, there is evidence to suggest that there are sex-specific associations between prenatal phthalate exposure and neurodevelopment. This has been reported as early as five days of age. Engel and colleagues [9] reported an association between maternal phthalate metabolites in urine and scores on the Brazelton Neonatal Behavioral Assessment Scale (BNBAS). The results showed that females, but not males, who had exposure to high molecular weight phthalates, had lower scores in orientation, and quality of alertness five days after delivery. Tellez-Rojo et al. [10] and Doherty et al. [6] observed associations between prenatal phthalate exposure and poorer neurodevelopment in females, but not males, between 2 and 3 years of age. In contrast, Kim and colleagues [5] showed an inverse association between prenatal phthalate exposure and neurodevelopment at six months of age in males, and Engel et al. [11] reported that prenatal phthalate exposure was associated with an increase in behavioral problems (greater aggression, poorer attention, more conduct problems) later in childhood in males but not females. While sex-specific associations have varied across studies, this might be due to variability in the age of assessment, the type of neurobehavioral outcome assessed, and/or the phthalates metabolites used as exposure biomarkers.

There are multiple pathways through which prenatal exposure to phthalates may disrupt the neurodevelopment of the developing fetus—for example through disruption of the hypothalamic-pituitary-thyroid (HPT) axis or the hypothalamic-pituitary-gonadal axis (HPG) [3,12,13]. Studies have found that higher exposure to phthalates is associated with a decrease in circulating thyroid hormones in pregnant women [14,15]. Maternal thyroid hormone during pregnancy has been shown to be essential for neurodevelopment [16]. In the case of the HPG axis, mechanistic studies support the potential for phthalates to act in an anti-androgenic manner leading to the disruption of normal sex-differentiation of the brain. In humans, this may be due to phthalate-associated reductions in testosterone production by the fetal testis [12,17,18,19]. Normally, there is an increase of testosterone in males between the 8th and 24th week of gestation [20]. Hence, this time-period has been identified as an important developmental window for impacts of endocrine disruptor exposure, particularly in the male fetus.

Although there is evidence to suggest there is a negative association between prenatal phthalate exposure and neurodevelopment, most previous studies have relied on a limited number of biospecimens collected at one or two time points during pregnancy to assess phthalate exposure, and have used assessments that capture global aspects of neurodevelopment rather than assessing specific cognitive domains. Given the short half-lives of phthalates and highly variable individual exposure even within a given day, a single biomarker sample is unlikely to reflect exposure levels across pregnancy. However, pooling samples collected throughout pregnancy is likely to provide a biomarker that is representative of average exposure across pregnancy [21,22,23,24,25]. In contrast, an individual sample collected between 8 and 24 weeks of gestation may be useful in the understanding sex-specific associations of prenatal phthalate exposure with cognitive outcomes, given the unique vulnerability (particularly of males) during this gestational window.

This study took advantage of research in developmental psychology, which typically uses infants’ looking behaviors as reliable and stable measures of specific aspects of cognition—in this case, physical reasoning. The physical reasoning task assesses young infants’ abilities to reason about conditions in which objects should remain in place versus when they should fall [26]. Furthermore, we created an automated version of the task that utilizes state-of-the-art infrared eye-tracking that allowed us to collect precise data in an efficient manner in a large number of infants. Hence, the present study aimed to characterize how prenatal exposure to phthalates (measured via urine metabolites in a pooled sample of five first morning urine samples collected across pregnancy and an individual sample collected at 16–18 weeks gestation) is associated with a specific cognitive domain, physical reasoning in 4.5-month-old infants.

## 2. Methods

### 2.1. Study Population

This study included pregnant women who were enrolled in the Illinois Kids Development Study (IKIDS) from January 2014 to August 2018. During their first prenatal visit at a local clinic in Urbana-Champaign, IL, USA the women received a brochure with information about the study and indicated whether they were interested in learning more. Women who expressed interest received a call from the research staff, during which the study was described in more detail and the woman’s eligibility to participate was ascertained. Eligible women were between 18 and 40 years of age, fluent in English, not in a high-risk pregnancy or carrying multiples, lived within a 30-min drive of the University of Illinois at Urbana-Champaign campus, and were not planning to move out of the area before their child reached one year of age. Women who reported use of over-the-counter or prescription medications were not excluded from the study. The study protocols were reviewed and approved by the Institutional Review Board at the University of Illinois at Urbana-Champaign. Women provided written informed consent at enrollment between 8 and 14 weeks of gestation. Subsequent to enrollment, study mothers participated in periodic assessments across pregnancy and after the child’s birth to collect health, diet, demographic, and lifestyle data as well as urine samples for exposure analysis.

### 2.2. Phthalate Exposure Assessment

First morning urine samples were collected at five time points across pregnancy (at approximately 10–14, 16–18, 22–24, 28–30, and 34–36 weeks of gestation). Samples were refrigerated from the time of collection (including during transport to the lab) until processing, which occurred within 24 h of collection. Specific gravity was measured using a handheld refractometer (Reichert TS 400) after allowing the samples to come to room temperature, and samples were aliquoted and stored at −80 °C using phthalate-free materials approved by the Centers for Disease Control and Prevention (CDC). A pooled sample was prepared by combining equal volumes of urine from all five time points, and specific gravity of the pooled sample was also measured.

Phthalate metabolites in the pooled urine sample and an individual urine sample collected at 16–18 weeks of gestation were measured at the CDC Division of Laboratory Sciences using high performance liquid chromatography-isotope dilution tandem mass spectrometry [27,28]. The CDC lab analyses have excellent sensitivity and reproducibility with coefficients of variation of 4–14%. For participants in this study, CDC measures included biomarkers of prenatal phthalate exposure for 14 phthalate metabolites from nine parent compounds. To account for urine dilution, all urine biomarker measures were adjusted for urine specific gravity [29].

This study focused on diethyl phthalate (DEP) and di-(2-ethylhexyl) phthalate (ΣDEHP) exposure because these two phthalates account for a large percentage of exposure in this cohort. In addition, diisononyl phthalate (DINP) was assessed as this is a common replacement for DEHP and exposure has been increasing [30]. Exposure to DEP was measured via its urinary metabolite, monoethyl phthalate (MEP). Exposure to DEHP was quantified as the molar sum of four major metabolites measured by the CDC—mono-2-ethyl-5-carboxypentyl phthalate (MECPP), mono-2-ethyl-5-hydroxyhexyl phthalate (MEHHP), mono-2-ethyl-5-oxohexyl phthalate (MEOHP), and mono-2-ethylhexyl phthalate (MEHP)—and expressed as ΣDEHP. DINP was quantified as the molar sum of two urinary metabolites: mono-(2,6- dimethyl-7-carboxyheptyl) phthalate (MCOP) and mono-isononyl phthalate (mNP) and expressed as ΣDINP. Women are never exposed to a single parent phthalate; hence, sums of multiple phthalates were also considered: the sum of anti-androgenic phthalates and the sum of all phthalates. The anti-androgenic sum (ΣAA) was composed of 11 urine metabolites identified based on evidence of phthalate-associated suppression of fetal testosterone production in animal models [31]. Exposure to the ΣAA was quantified as the molar sum of the metabolites: monoisobutyl phthalates (miBP), monobenzyl phthalate (mBzP2), mono-2-ethylhexyl phthalate (MEHP), mono-2-ethyl-5-hydroxyhexyl phthalate (MEHHP), mono-2-ethyl-5-oxohexyl phthalate (MEOHP), mono-2-ethyl-5-carboxypentyl phthalate (MECPP), mono-(2,6- dimethyl-7-carboxyheptyl) phthalate (MCOP), mono-isononyl phthalate (mNP), mono-n-butyl phthalate (mBP), mono-hydroxyisobutyl phthalate (mHiBP), and mono-hydroxybutyl phthalate (mHBP). For the ΣAA, metabolites of DINP were weighted less than other metabolites because mechanistic laboratory animal studies found DINP to have less potent anti-androgenic effects [32,33]. For the sum of all phthalates (Σall phthalates), the molar sum of the 14 metabolites measured in all of the women was used: monoisobutyl phthalates (miBP), monobenzyl phthalate (mBzP2), mono-2-ethylhexyl phthalate (MEHP), mono-2-ethyl-5-hydroxyhexyl phthalate (MEHHP), mono-2-ethyl-5-oxohexyl phthalate (MEOHP), mono-2-ethyl-5-carboxypentyl phthalate (MECPP), mono-(2,6- dimethyl-7-carboxyheptyl) phthalate (MCOP), mono-isononyl phthalate (mNP), mono-n-butyl phthalate (mBP), mono-hydroxyisobutyl phthalate (mHiBP), and mono-hydroxybutyl phthalate (mHBP), monocarboxy-isononyl phthalate (MCNP), and mono-(3-carboxyl propyl) phthalate (MCPP).

### 2.3. Physical Reasoning Task

**Figure 1:**
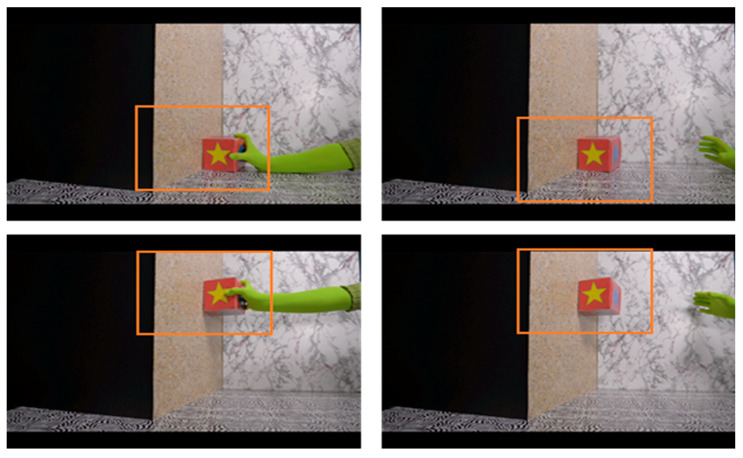
The physical reasoning task consists of two video events, the possible (**top**) and the impossible (**bottom**) event. The orange squares encompass the interest area used to identify infants who are looking at the box. Re-printed with permission from Merced-Nieves et al. (2020). Association of prenatal maternal perceived stress with a sexually dimorphic measure of cognition in 4.5-month-old infants.

The task has been described in more detail in a previous publication [34]. Briefly, this is an automated version of the physical reasoning task designed by Baillargeon [26]. Study infants were assessed at 123–146 days of age; hereafter, referred to as the infant’s 4.5-month visit. In this automated version, study infants were seated on a parent’s lap while watching short videos on a large screen high-definition television. In addition, mothers were asked to wear darkened sunglasses and to remain silent during the test to prevent where they looked or what they said from influencing the infant’s looking behavior. Infant looking behaviors were tracked using an EyeLink 1000 Plus infrared eye tracker (SR Research Ltd., Mississauga, Ontario, Canada). The physical reasoning task consisted of two video events: a possible event and an impossible event. In the possible event, a gloved hand came into the scene holding a box. The hand slid the box along the floor and placed it against a wall and released it briefly before withdrawing the box out of the scene. In the impossible event, the same gloved hand placed the box against the wall midair, leaving the box suspended without support underneath. Each event lasted for about 8.5 s and was repeated eight times for a total of 68 s. Since eye-tracking allows for a fine-grained analysis of looking responses, we were able to define an area of interest (Figure 1) and measure infants’ looking time within this area. To ensure that infants were attending to the task, we established a minimum looking time in the interest area of 7.5 s for each event, which was twice the length of time the box remained in the interest area each time the glove released it. Half of the infants saw the impossible event first and the other half saw the possible event first, and the order in which infants saw the events was randomized in blocks of 48 infants within each sex. Physical reasoning ability was measured by calculating the difference in total looking time between the impossible and possible events (impossible minus possible) wherein a higher number means the infant looked longer at the impossible than the possible event. At 4.5 months of age, females typically look significantly longer at the impossible than at the possible event, suggesting that they expect the unsupported box to fall and are surprised when it does not. Males tend to look equally at the two events suggesting that they do not share this expectation. Males typically develop this expectation 4–6 weeks later, at around 5.5 months of age [26].

### 2.4. Statistical Analysis

All statistical analyses were performed using SAS 9.4 software (SAS Institute Inc., Cary, NC, USA). Regression diagnostics generally supported the use of continuous, untransformed biomarker measures as well as the use of linear models to characterize hypothesized associations. Multivariable linear regression models were used to examine the association between an interquartile range (IQR) increase in each continuous maternal urinary biomarker of exposure (ΣDEHP, ΣDINP, MEP, ΣAA, and the Σall phthalates) and looking time difference (in seconds). Potential covariates for inclusion in models were identified based on a priori knowledge, a directed acyclic graph [35], and additional bivariate analyses (spearman correlations when both variables were continuous and ANOVA when one of the two variables was categorical). These analyses assessed the association between each covariate and each exposure measure and each covariate and the outcome. These potential covariates were maternal age at birth, education, IQ, and parity; household income; infant’s age at assessment, sex, and order of event presentation (possible first or impossible first). Only the order of event presentation and infant sex were associated with the outcome. Maternal age was weakly correlated with MEP exposure biomarkers (r_s_ = −0.13 and −0.15, 16–18 weeks and pooled, respectively). Hence, final models included covariates for order of event presentation, infant sex and maternal age.

Initially, models assessing the overall effect of exposure were considered. Main effects of exposure were considered significant if *p*-values were less than 0.05. Based on previous findings of phthalate effect modification by infant’s sex, the interaction of sex by exposure was also assessed. Finally, we also explored potential additional interactions, including of exposure with order of event presentation. Specifically, two-way interactions between exposure and order of event presentation and between sex and order of event presentation were assessed, and models including all two-way and three-way interactions among these three variables were also evaluated. Interactions found to have a *p*-value > 0.10, were removed from the model.

A number of sensitivity analyses were performed to assess the robustness of any observed associations. Regression diagnostics were performed to identify potential influential points, defined as observations that had extreme Cook’s Distance (D > 0.1); sensitivity analyses were done with influential observations removed. Among the women in our final analyses, there were few smokers (n = 3 or 4, varied by model) and relatively few women reporting first trimester alcohol consumption (n = approximately 30, varied by model). Hence, we assessed potential confounding by early pregnancy smoking or alcohol intake in secondary analyses removing women who reported any smoking and by adjusting for first trimester alcohol intake. We also ran models that included additional demographic variables beyond those included in the primary models, including maternal education, household income, and infant’s age at time of testing. Finally, we ran a model adjusting for maternal prenatal stress (categorized as: low, medium, or high with high as the reference value).

## 3. Results

### 3.1. Descriptive Data

Between 2014 and 2018, 558 mother-infant pairs were enrolled in IKIDS, 481 of whom had biomarkers of prenatal phthalate exposure available, A total of 386 of those (80%) participated in the 4.5-month infant evaluation, but the physical reasoning task was only administered to 284 of those infants. Of those, a total of 159 infants (78 females and 81 males) met criterion (minimum looking time of 7.5 s) on the task, had both phthalate and covariate data available, and were included in these analyses. Table 1 details the characteristics of the sub-sample of mothers whose infants were included in the analysis compared to those in the full IKIDS cohort and the subsample with phthalate data available. There were differences in proportion of mothers who reported any smoking and drinking during the first trimester. Specifically, compared to the full cohort or those with phthalate data, a smaller percentage of the women included in this analysis reported drinking or smoking. In addition, there was a difference in the proportion of males and females in the full cohort. However, this difference was no longer present after subsetting on the proportion of the sample with non-missing information. In the study subsample, at the time of enrollment, more than 80% of the mothers had at least a college degree and 69% had an annual household income of $60,000 per year or greater. Maternal smoking and alcohol consumption data were only available for the first trimester. Only 4% of the subsample included in these analyses reported any smoking during the first trimester and about 23% reported any alcohol consumption. Alcohol consumption included recollections from weeks 0–14 meaning some of the drinking reported was before knowledge of the pregnancy. In addition, most women who did drink reported less than 4 drinks per week. All infants in this subsample were born full-term (37 weeks of gestation or later) with an average gestational age of 39.5 weeks; 22% were delivered via cesarean section.

### 3.2. Exposure Characterization

A summary of pregnancy urine biomarker concentrations reflecting prenatal phthalate exposures for infants included in the current analysis is shown in Table 2. Biomarker concentrations for this subset were very similar to those for the full cohort and to the latest reported biomonitoring data for reproductive age women from the National Health and Nutrition Examination Survey (NHANES) [36].

### 3.3. Phthalates and Physical Reasoning

**Figure 2:**
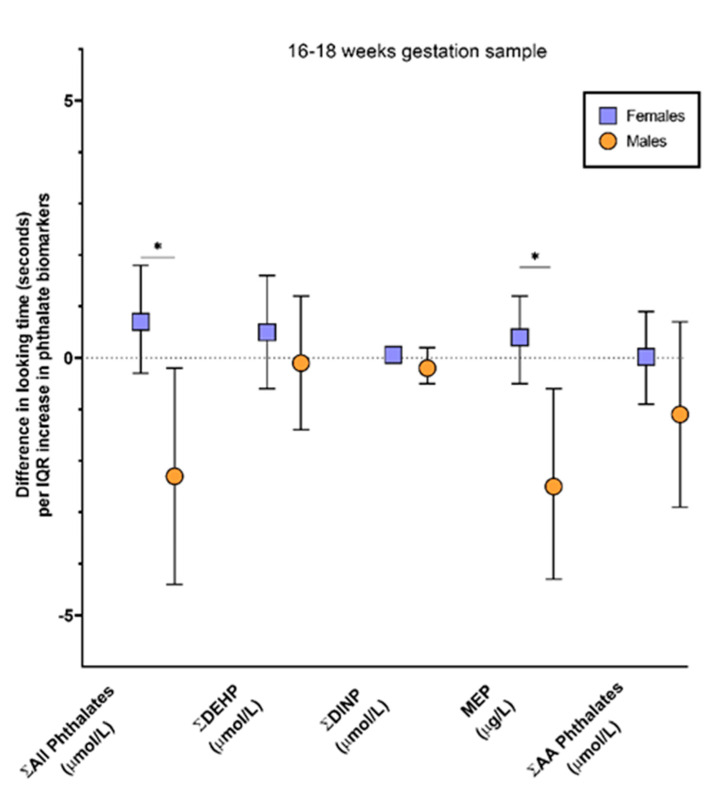
Association between biomarkers of prenatal phthalate exposure in maternal urine at 16–18 weeks of gestation and difference in looking time (impossible event looking time—possible event looking time, seconds) on a physical reasoning task in 4.5-month-old IKIDS infants. Associations are based on an interquartile range (IQR) increase in each specific gravity adjusted exposure biomarker with models adjusted for maternal age at birth, infant sex, order of event presentation (impossible first or possible first), and sex by exposure biomarker. * interaction *p*-value < 0.05.

**Figure 3:**
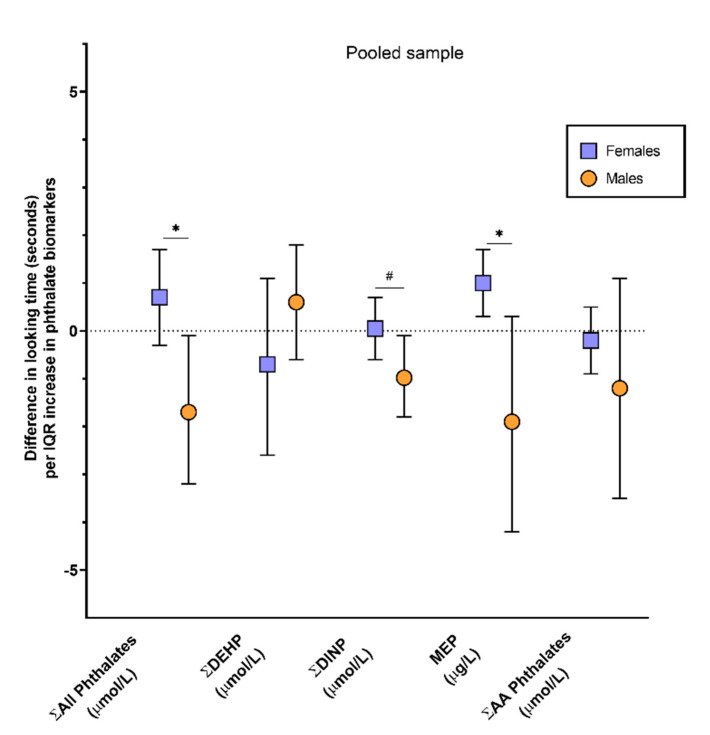
Association between biomarkers of prenatal phthalate exposure in a pool of maternal urines collected across pregnancy and difference in looking time (impossible event looking time—possible event looking time, seconds) on a physical reasoning task in 4.5-month-old IKIDS infants. Associations are based on an IQR increase in each specific gravity adjusted exposure biomarker with models adjusted for maternal age at birth, infant sex, order of event presentation (impossible first or possible first) and sex by exposure biomarker. * interaction *p*-value < 0.05, # interaction *p*-value < 0.10.

Simple models exploring main effects of phthalate exposure did not reveal any significant effects. In models exploring potential modification of exposure effects by infant sex or order of event presentation, there was no evidence of significant (*p* < 0.10) two-way or three-way interactions among these measures except for exposure by sex interactions for a subset of exposure biomarkers. Hence, the results presented here focus on simplified models that include model covariates and the sex by exposure interaction term. Results of covariate-adjusted general linear models, for each phthalate of interest at 16–18 weeks gestation and for the pooled sample are presented in Table 3. In models examining the 16 to 18-week time point, there were significant sex by exposure interactions for MEP (*p*-value = 0.007) and Σall phthalates (*p*-value = 0.01). An IQR increase in MEP or the Σall phthalates was associated with a negative looking time difference in males (β = −2.5; 95% CI: −4.4, −0.6; *p*-value = 0.01 and β = −2.3; 95% CI: −4.4, −0.2; *p*-value = 0.03, respectively), but no differences in looking times for females (Figure 2 and Table 3). In the models examining associations with phthalate measures in the pooled sample, there were also sex by exposure interactions for MEP (*p*-value = 0.02) and the Σall phthalates (*p*-value = 0.01) as well as for ΣDINP (*p*-value = 0.06). An IQR increase in MEP was associated with a 1.0 s (95% CI: 0.3, 1.7; *p*-value = 0.04) increase in looking time difference in females, whereas, a non-statistically significant negative looking time difference was seen in males (β = −1.9; 95% CI: −4.4, 0.3; *p*-value = 0.09). An IQR increase in ΣDINP or Σall phthalates was associated with a negative looking time difference in males (β = −1.0; 95% CI: −1.8, −0.1; *p*-value = 0.03 and β = −1.7; 95% CI: −3.2, −0.1; *p*-value = 0.03, respectively) but, again, these phthalates were not associated with differences in looking time in females (Figure 3 and Table 3). As shown on Table 3, no other statistically significant associations were observed.

### 3.4. Sensitivity Analyses

Findings were essentially unchanged when we added additional demographic variables to our models (Table S1), excluded infants whose mothers reported smoking during the first trimester (Table S2), or adjusted for maternal prenatal stress (Table S3). When we adjusted models for maternal alcohol consumption during the first trimester (Table S4), results were similar with one exception. We observed a sex by exposure interaction for the anti-androgenic sum in the pooled sample (*p*-value= 0.05) which wasn’t observed in our main models. An IQR increase in ΣAA was associated with a negative looking time difference in males (β = −3.0; 95% CI: −5.7, −0.3; *p*-value = 0.03), but was not associated with looking time differences in females (Table S4).

## 4. Discussion

Our study showed that prenatal exposure to phthalates is associated with sex-specific changes in performance on the physical reasoning task at 4.5 months of age. Results showed that higher prenatal exposure to MEP (16–18 weeks of gestation and pooled sample), ΣDINP (pooled sample), and the Σall phthalates (16–18 weeks gestation and pooled sample) were each associated with male infants looking longer at the possible event than the impossible event. Together these findings suggest a potential adverse impact of phthalates on physical reasoning in males. Previously reported sex-specific associations of phthalates with neurodevelopment have varied with some studies reporting effects in males [6,9,10] and others in females [5,11]. Interestingly, even though we observed adverse associations in males but not females, these findings do not seem to correlate with anti-androgenic activity as the most consistent results were observed for exposure to MEP, ΣDINP, and Σall phthalates, not the ΣAA phthalates. Thus, the mechanism for this sex-specific association remains unclear. It is important to note, we saw effects of ΣDINP which is used as a common replacement for ΣDEHP, and not of ΣDEHP itself. This adds to the growing body of evidence for “regrettable replacements”, where the replacement chemical has the potential to be more detrimental than the original. Another important thing to note is that there were consistent findings for the single time point at 16–18 weeks and the pooled sample. Previous literature has raised the issue of exposure misclassification when a single urine sample is used to estimate phthalate exposure, given the short half-lives of phthalates and highly variable individual exposure across days and even within a given day. However, in this case, we did not find that there was a difference.

Previous research has shown that 4.5-month old males tend to look equally at the two events in this physical reasoning task. Whereas, females of the same age and slightly older males (4–6 weeks older) look significantly longer at the impossible than the possible event [26]. Baillargeon argues that these different responses reflect differences in infants’ knowledge about what should happen when an object is unsupported (impossible event). This knowledge is acquired through their everyday observations of the world. Infants who have acquired this knowledge see the possible event as familiar because it matches their everyday observations, and they see the impossible event as novel because it violates their expectations. Infants who are in the process of acquiring this knowledge cannot detect the violation in the impossible event, even if they can recognize the familiarity of the possible event. Interestingly, research has also shown that in the very initial stages of learning, infants may prefer a familiar stimulus over a novel one [37]—like the males with higher phthalate exposure in our study. The interpretation is that this is an adaptive mechanism by focusing on familiar stimuli, infants can learn (make sense of it) faster before they turn their attention to novel stimuli. Thus, in our current study, phthalate exposure appears to be slowing down males in their process of acquiring knowledge about supported and unsupported objects. However, further studies with older males are needed to test this possibility.

Unexpectedly higher prenatal exposure to MEP (pooled sample) was associated with females looking longer at the impossible than the possible event. This result suggests an improvement in physical reasoning in the females. There are other studies in the literature that have found associations between higher phthalate exposure and better neurodevelopmental outcomes [18]. In addition, there is evidence to suggest that endocrine disrupting chemicals can have non-monotonic dose response curves so it is conceivable that there would be a range of MEP exposure that is associated with better outcomes [38]. Alternatively, residual confounding could explain an unexpected beneficial association.

There are some strengths and limitations to this study that should be considered when evaluating its findings and implications. This study took advantage of urine samples pooled from multiple samples collected across pregnancy, providing a measure of average exposure throughout pregnancy. Furthermore, we also evaluated the associations with the sample collected between 16 and 18 weeks of gestation, which is an important window in the sexual differentiation of the brain. Another important strength was the use of state-of-the-art eye tracking technology that allowed automated collection of precise looking behavior at a very early age. A potential limitation is the small sample size; however, our findings were consistent across sensitivity analyses suggesting robust associations. Finally, due to the small sample size we were unable to utilize more complex statistical methods for exposure mixtures analysis (e.g., weighted quantile sum regression or Bayesian Kernel Machine Regression) to assess the effects of phthalate mixtures; future analyses in larger samples should evaluate this approach.

## 5. Conclusions

In conclusion, the findings presented here suggest that prenatal exposure to phthalates (including exposure to a common DEHP substitute, DINP) is associated with poorer physical reasoning in males at 4.5 months of age. Our findings provide additional evidence that prenatal phthalate exposure can impact early cognitive development and support the potential for there to be sex differences in sensitivity to the impact of phthalates on cognition.

## Figures and Tables

**Table 1:** Demographic and lifestyle characteristics of mother-infant pairs enrolled in IKIDS between 2014–2018 and those included in the analysis.

		Full Cohort (n = 558)		Participants with Phthalate Data (n = 481)		Current Analysis (n = 159)	
		N (%)	Mean (SD)	N (%)	Mean (SD)	N (%)	Mean (SD)
**Maternal race**							
White, Non-Hispanic		446 (79.9)		388 (80.7)		127 (80)	
Other		111 (19.9)		92 (19.1)		32 (20)	
Unknown/Missing		1 (0.2)		1 (0.2)			
**Maternal age (years)**			30.2 (4.3)		30.3 (4.1)		30.4 (3.9)
**Maternal education**						
Some college or less		123 (22.0)		90 (18.7)		29 (18)	
College degree or higher		435 (78.0)		391 (81.3)		130 (82)	
**Annual household income**						
<$60,000		171 (30.6)		138 (28.7)		48 (30)	
≥$60,000		382 (68.5)		340 (70.7)		109 (69)	
Unknown/Missing		5 (0.9)		3 (0.6)		2 (1)	
**Maternal smoking ^1^**		30 (5.4)		23 (4.8)		6 (4)	
**Maternal drinking ^2^**		225 (40.3)		199 (41.4)		37 (23)	
**Mode of delivery (% cesarean)**		127 (22.8)		125 (26)		34 (22)	
**Gestational age (weeks)**			39.3 (1.5)		39.3 (1.4)		39.5 (1.1)
**Infant sex**							
Female		237 (42.5)		247 (51.2)		78 (49)	
Male		259 (46.4)		234 (48.7)		81 (51)	
Unknown/Missing		62 (11.1)		0 (0)		0 (0)	

^1^ Any smoking during the first trimester of pregnancy (yes/no). ^2^ Any drinking during the first trimester of pregnancy (yes/no).

**Table 2:** Characterization of exposure biomarkers used in analyses (specific gravity adjusted) for infants included in current analysis.

		16–18 Weeks Gestation Sample (n = 158)				Pooled Sample (n = 159)			
Exposure ^1^	Units	Median	IQR ^2^	Minimum	Maximum	Median	IQR	Minimum	Maximum
MEP	μg\L	24.34	33.09	0	576.43	29.05	32.62	4.8	672.76
Σ DEHP	µmol/L	0.06	0.05	0.01	0.76	0.07	0.05	0.03	0.5
Σ DINP	µmol/L	0.02	0.04	0.002	2.97	0.03	0.04	0.01	0.82
Σ AA	µmol/L	0.21	0.18	0.03	2.97	0.23	0.17	0.09	4.17
Σ All phthalates	µmol/L	0.43	0.42	0.08	3.83	0.50	0.38	0.14	5.42

^1^ Abbreviations: MEP, monoethyl phthalate; Σ DEHP, di-(2-ethylhexyl) phthalate; Σ DINP, diisononyl phthalate; Σ AA, anti-androgenic sum; Σ All phthalates, sum of all phthalate metabolites measured. ^2^ Abbreviation: IQR, interquartile range

**Table 3:** Associations between biomarkers of prenatal phthalate exposure and physical reasoning among 159 IKIDS infants assessed at 4.5 months.

		Main Effect		Females (n = 78)		Males (n = 81)	
Exposure Measure	N	β Estimate	95% Confidence Interval	β Estimate	95% Confidence Interval	β Estimate	95% Confidence Interval
Phthalates (16–18 weeks gestation sample) ^1^							
MEP (μg\L)	158	0.3	−0.3, 0.9	**0.4**	**−0.5, 1.2**	−2.5	**−4.4, −0.6**
Σ DEHP (µmol/L)	158	0.2	−0.6, 1.1	0.5	−0.6, 1.6	−0.1	−1.4, 1.2
Σ DINP (µmol/L)	158	0.004	−0.2, 0.2	0.06	−0.1, 0.2	−0.2	−0.5, 0.2
Σ Anti-Androgenic (µmol/L)	158	−0.2	−1.0, 0.6	0.02	−0.9, 0.9	−1.1	−2.9, 0.7
Σ All phthalates (µmol/L)	158	0.2	−0.8, 1.1	**0.7**	**−0.3, 1.8**	−2.3	**−4.4, −0.2**
Phthalates (Pooled sample)							
MEP (μg\L)	159	0.3	−0.3, 0.8	**1**	**0.3, 1.7**	**−1.9**	**−4.2, 0.3**
Σ DEHP (µmol/L)	159	0.2	−0.8, 1.2	−0.7	−2.6, 1.1	0.6	−0.6, 1.7
Σ DINP ^2^ (µmol/L)	158	−0.3	−0.7, 0.2	**0.05**	**−0.6, 0.7**	−1	**−1.8, −0.1**
Σ Anti-Androgenic (µmol/L)	159	−0.3	−1.0, 0.4	−0.2	−0.9, 0.5	−1.2	−3.5, 1.1
Σ All phthalates (µmol/L)	159	0.04	−0.8, 0.9	**0.7**	**−0.3, 1.7**	**−1.7**	**−3.2, −0.1**

^1^ Phthalate metabolites for the 16–18-week urine were not available for one observation. ^2^ Model had a potential influential point removed (defined as Cook’s D values > 0.1). Values bolded were sex by exposure interaction was statistically significant (*p*-value < 0.05).

## Data Availability

The data presented in this study are available on request from the corresponding author. The data are not publicly available due to privacy or ethical restrictions.

## Supplementary Materials

The following are available online at https://www.mdpi.com/1660-4601/18/4/1838/s1, Table S1: Sensitivity analyses examining associations1 between biomarkers of prenatal phthalate exposure and physical reasoning2 among 159 IKIDS infants assessed at 4.5 months. Models additionally adjusted for maternal education, household income and infant age at testing, Table S2: Sensitivity analyses examining associations1 between biomarkers of prenatal phthalate exposure and phys-ical reasoning2 among 159 IKIDS infants assessed at 4.5 months. Models excluded mothers who reported any smoking during the first trimester of pregnancy, Table S3: Sensitivity analyses examining associations1 between biomarkers of prenatal phthalate exposure and physical rea-soning2 among 159 IKIDS infants assessed at 4.5 months. Models additionally adjusted for ma-ternal prenatal stress, Table S4: Sensitivity analyses examining associations1 between biomarkers of prenatal phthalate exposure and physical reasoning2 among 159 IKIDS infants assessed at 4.5 months. Models additionally adjusted for maternal alcohol intake during the first trimester of pregnancy.
